# Acute toxic effects of areca nut on central nervous system and liver: A case report

**DOI:** 10.1002/ccr3.7976

**Published:** 2023-10-04

**Authors:** Seyede Maryam Mahdavi Mortazavi, Maryam Ataollahi, Amirali Mashhadiagha, Seyed Ali Moosavi, Reze Moshfeghinia, Marzieh Soheili

**Affiliations:** ^1^ Department of Pediatrics, School of Medicine, Namazi Teaching Hospital Shiraz University of Medical Sciences Shiraz Iran; ^2^ Department of Pediatrics, School of Medicine, Namazi Teaching Hospital, Abu Ali Sina Center for Medicine & Organ Transplant Shiraz University of Medical Sciences Shiraz Iran; ^3^ Student Research Committee Shiraz University of Medical Sciences Shiraz Iran; ^4^ College of Pharmacy and Health Sciences Western New England University Springfield Massachusetts USA

**Keywords:** an addictive substance, Areca nut, case report, hepatotoxic agent, hepatotoxicity

## Abstract

Areca nut (AN) is an addictive substance widely used in different world regions. There are several side effects associated with the use of AN, which have already been reported. However, the reports on hepatotoxicity of AN are sporadic and non‐conclusive. In the present case report, we investigated the hepatotoxicity of AN in a four‐year‐old Iranian girl who was transferred to our medical center with abdominal pain, vomiting, diarrhea, fever, and other symptoms such as hematuria, decreased mental status, multiple seizure episodes. After a comprehensive evaluation, it was concluded that these signs and symptoms were all attributed to AN consumption, which was given by her mother to control diarrhea. Eventually, the patient medical conditions were managed successfully, and she survived by intense medical care. In conclusion, we suggest AN should be considered a potential hepatotoxic agent.

## Graphical abstract

Is brought.

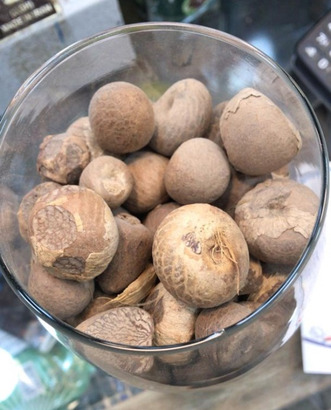

## 1 BACKGROUND

Areca nut (AN) is consumed by 600 million people worldwide, and betel quid, a preparation containing AN, is consumed by 10%–20% of the world's population. The world's largest consumers of AN are India and other Southeast Asian countries.^1^ After nicotine, ethanol, and caffeine, AN is the most commonly used addictive substance globally. There is strong evidence that AN, whether with or without tobacco use, can cause cancer.^2^ According to the IARC, the AN is carcinogenic in humankind. It has been linked to cancers in different body areas such as the mouth, pharynx, esophagus, liver, bile ducts, and the uterus.^3^ This report describes a child's medical condition transferred to our medical center with altered mental status, seizure, elevated hepatic transaminases, hematuria, and hemolysis associated with AN toxicity.

## 2 CASE PRESENTATION

**FIGURE 1:**
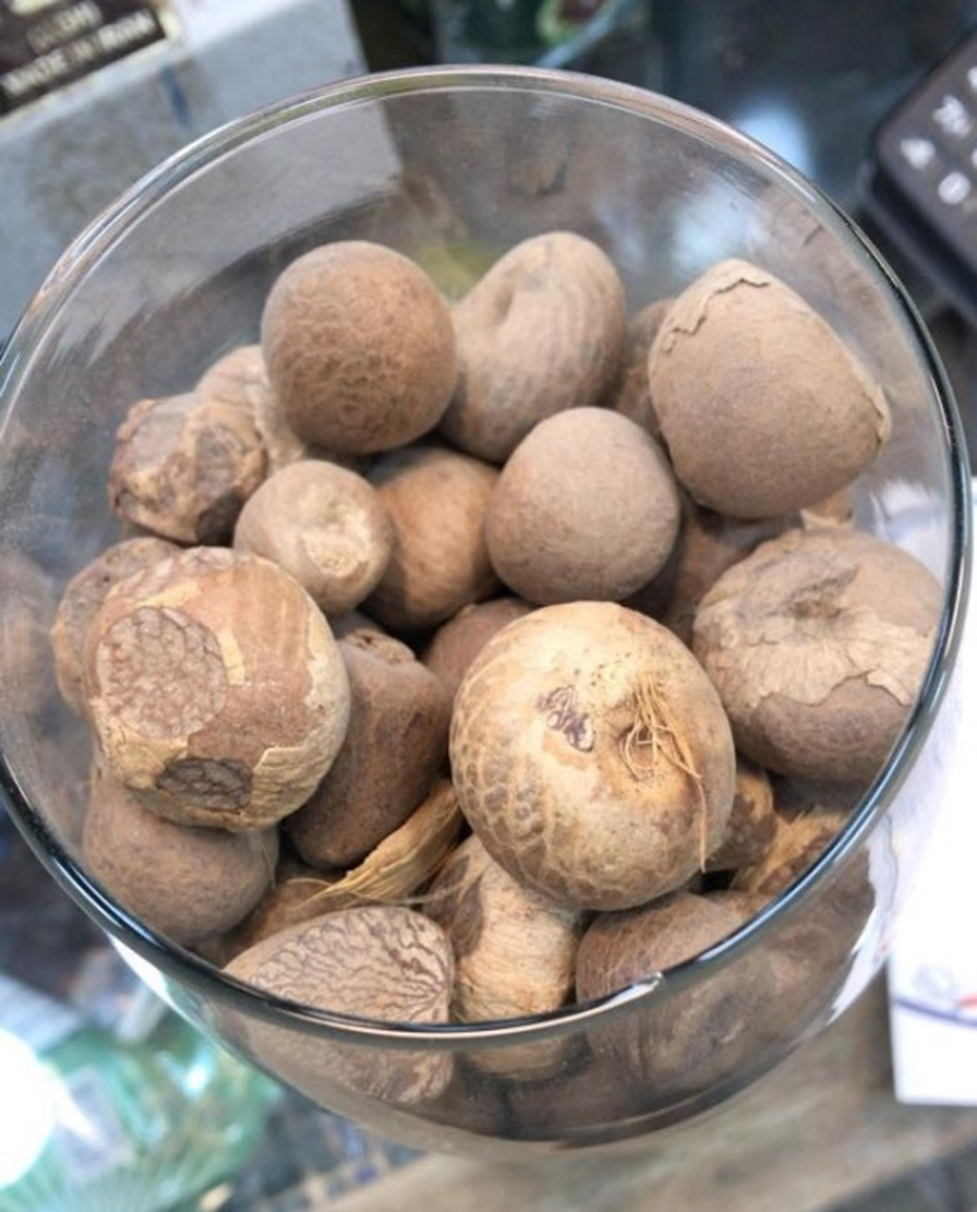
Areca nut.

A four‐year‐old girl was referred to our hospital with hematuria and altered mental status, followed by frequent vomiting, diarrhea, and fever episodes. After talking to her mother, it was indicated that her general health status was excellent 2 days before her admission into the medical center. There was no remarkable health condition in her medical history. Her body weight was 12 kg. In the past 2 days, she developed a sudden abdominal pain, diarrhea, nausea, and three episodes of vomiting about 6 h after her meal. Before admission to the emergency department of Jahrom Hospital (Jahrom, Iran), she was given Ibuprofen syrup (3 mL) for her fever and AN (Figure 1) powder (two tablespoons) for her diarrhea by her mother. The patient's condition deteriorated immediately after AN ingestion. She developed a progressive paleness, and her extremities turned cold. She had blood in her urine (gross hematuria) and two episodes of generalized tonic–clonic seizure with an interval of 30 min. The duration of the first seizure episode was about 10 min, and the second episode lasted for 3 min. Eventually, she was brought to the ED of Jahrom hospital. During the initial evaluations in the ED, she was febrile, pale, and had tachycardia, gross hematuria, and non‐exudative conjunctivitis. Her heart rate was 140 beats/min, and her respiratory rate was 40/min. Oxygen saturation was 85%, blood pressure was 90/50 mmHg, and her body temperature was 39.4°C.

Considering the patient's critical condition and lack of a PICU in Jahrom hospital, the medical team transferred her to Namazee Hospital, Shiraz, Iran. Due to high suspicion of a septic shock, intravenous hydration, and Vancomycin (15 mg/kg i.v. Q8H), and Ceftriaxone (100 mg/kg i.v. Q6h) were empirically prescribed. The patient was promptly transferred to PICU with GCS 13/15. At the PICU, rigorous intravenous hydration and the administration of previous antibiotics continued, and acyclovir (10 mg/kg i.v. Q8h) was started to treat possible herpetic encephalitis. Additionally, after pediatric neurology consultation, an anticonvulsant, Levetiracetam (10 mg i.v. Q12h), was administered.

Further evaluations for neurological symptoms were conducted, including biochemical and microbiological examination of CSF and brain computed tomographic scan. The results of these tests were unremarkable. Electroencephalography showed some paroxysmal sharp waves, contrary to a simple febrile seizure. Thus, intravenous acyclovir was stopped after neurology consultation.

Initial laboratory results showed normal electrolyte levels except for hypokalemia, high values of CRP (>150 mg/L), LDH (3570 U/L), and fibrinogen (611 mg/dL). Besides, the negative coombs' test and sufficient activity of G6PD all indicated non‐immune hemolysis. Venous blood gas assessment included pH: 7.43, PCO_2_: 14.3 kPa, and HCO_3_: 9.6 mEq/L. Echocardiography was normal. Renal function tests were within the normal range (BUN 14 mg/dL, Creatinine 0.42 mg/dL), which ruled out the hemolytic uremic syndrome followed by *Escherichia coli* gastroenteritis. Elevated hepatic transaminases AST: 2670, ALT: 970 U/L, and INR: 1.76 were observed. Total and direct bilirubin: 0.7, 0.3 g/dL, and alkaline phosphatase: 296 IU/L were reported within the normal range. Urine analysis showed +4 blood and +2 albumin, which could be due to hemolysis and fever. There was +3 occult blood and a high white blood cell count in the stool examination. Virology investigations were negative for HBV, HCV, HAV, SARS‐Cov‐2, and HSV 1 and 2 (Table 1).

**TABLE 1:** Laboratory evaluation on the first and last days of admission (NC, not checked; NR, not reported).

	First day of admission	On the third day of admission	Last day of admission		First day of admission	On the third day of admission	Last day of admission
Hemoglobin (Hb)	10.2	8.7	10.4	Bilirubin Total	0.7	0.2	0.3
Hematocrit	31.8	27.3	27.3	Bilirubin Direct	0.3	0.1	0.1
MCHC	32.1	31.9	30.0	ALP	296	320	363
MCV	82.2	87.3	87.8	CPK	173	3215	1250
WBC count	6.6	12.8	9.7	LDH	3570	NC	5510
Neutrophil (Seg)%	68.4	NC	47.1	Ferritin	209	NC	NC
Lymphocyte (%)	22.2	NC	43.4	Lactate	19	NC	NC
Eosinophil	NR	NC	3	CRP	>150	13	4
Platelet count	243	301	526	Coombs+	Negative	NC	NC
RDW	13.5	13.2	13.2	Indirect Coombs	Negative	NC	NC
Retic count	0.5	NC	1.5	Urine analysis			
RBC count	3.87	3.28	3.11	Specific Gravity	1.014	1.004	1.009
MCH	26.4	26.5	26.4	Glucose	Negative	Negative	Negative
PDW	10.3	9.8	10.5	Bilirubin	Negative	Negative	Negative
Mixed (%)	9.4	NC	9.8	Ketone	Trace	Negative	Negative
MPV	8.5	9.1	8.8	Blood	4+	Negative	Negative
P‐LCR	14.4	NC	15.6	Ascorbic acid	Negative	Negative	Negative
Monocyte %	7	NC	NC	Nitrite	Negative	Negative	Negative
ESR 1	36		67	Alb	3+	Negative	Negative
PT	23.8	14.4	15.6	RBC	1–2	0–1	0–1
INR	1.76	1.07	1.16	WBC	0–1	2–3	6–8
PTT	41.8	30.3	31.1	Bacteria	Few	Rare	Few
G6PD	Sufficient	NC	NC	Crystal	Many	NC	NC
Fibrinogen level	611		311	Appearance	Turbid	Clear	Clear
BUN	14	8	15	pH	Acid	Alkaline	Alkaline
Cr	0.42	0.43	0.44	Color	Brown	Yellow	Yellow
K	3.8	4.2	4.7	Leukocyte esterase	Negative	Negative	Negative
Na	136	140	141	Stool Exam			
Glucose (serum)	126	NC	80	Consistency	Mucoid	Soft	Soft
Calcium total	0.42	9.5	10.1	Stool OB	3+	Trace	Trace
Total protein	8.6	4.5	5.8	Stool WBC	Many		NR
Albumin	3.1	3.2	3.8	Stool fat quantitative–acidified	Not seen	Not seen	Not seen
AST	2670	920	62	Blood culture	No growth	NC	No growth
ALT	970	750	257	Urine culture	No growth	NC	No growth
TG	202	NC	NC	CSF analysis			
Chol	162	NC	NC	Total cell	10	NC	NC
HSV PCR	Not detected	NC	NC	RBC count	10	NC	NC
ABG assessment				CSF culture	Negative	NC	NC
pH	7.43		7.53	–	–	–	–
PCO_2_	14.3		28.9	–	–	–	–
HCO_3_	9.6		24.5	–	–	–	–
BE	−11		1–9	–	–	–	–
PO_2_	152.5		77.8	–	–	–	–

On the second day of PICU admission, 120 mL packed cells were transfused due to the decreased hemoglobin level (initial value: 10.2 g/dL, follow‐up value: 9 g/dL). All the patient's signs and symptoms were alleviated gradually. On the sixth day of admission, the patient was transferred to the pediatric GI ward after stabilizing her vital signs, decreasing the levels of AST and ALT, and normal ABG and imaging. While she was admitted to the GI ward, antibiotic therapy, and Levetiracetam administration were continued. Zinc Sulfate (10 mg, p.o. daily), ursodeoxycholic acid (130 mg p.o. BID), and folic acid (5 mg daily) were added to her treatment. Eventually, The patient was discharged in a stable situation. The laboratory tests were repeated after 1 month, and all the results were within the normal range.

## 3 DISCUSSION AND CONCLUSION

AN is an addictive substance widely used in South and Southeast Asia and the Asia Pacific region, particularly among migrant communities in Africa, and North America.^4^ If consumed in large quantities, AN can cause acute toxic symptoms such as dyspnea, tachypnea, tachycardia, palpitations, hypotension, chest tightness, nausea, vomiting, dizziness, abdominal colic, and even myocardial infarction and coma; however, in the majority of cases, the effects are transient, and patients recover quickly.^6^

In the study reported by Sundqvist et al., the AN extract decreased cell survival and membrane integrity in a dose‐dependent manner. The ANs aqueous extract was highly cytotoxic and genotoxic and resulted in the formation of both DNA single‐strand breaks and DNA protein cross‐links.^5^ Another study indicated that tumors could arise in the buccal mucosa after exposure to AN.^6^ Furthermore, the AN showed cytotoxic effects on red blood cells, resulting in hemolysis, hematuria, and anemia.^7^

In addition to AN being carcinogenic to the oral cavity, pharynx, esophagus, liver, and uterus, numerous adverse effects on the entire body organs have been reported. AN causes euphoria, tachycardia, hypertension, GABA inhibition, and neuronal damage while not affecting concentration or memory. It results in hyperlipidemia, vasospasm, and cardiac arrhythmias, increasing the risk of myocardial ischemia. Chronic consumption of AN results in hypothyroidism, prostate hyperplasia, infertility, and vitamin D deficiency.^2^ Additionally, AN has been shown to increase the rate of preterm birth and stillbirth in tobacco smoker women.^8^ Besides, in the review of Javed et al., obesity, increased waist circumference, elevated triacylglycerol level, metabolic syndrome, hyperglycemia, and Type 2 diabetes were reported as the side effects of AN.^9^

In our current case report, hepatotoxicity was one of the clinical findings. Because of the patient's unstable hemodynamics, the hepatic shock was considered one of the differential diagnoses for elevated liver function. Various medical conditions cause hepatic shock, including hemorrhage, surgery, respiratory failure, infection, and chronic shock. Other causes include failure of microcirculation, a systemic inflammatory reaction, and adverse treatment effects in patients admitted into the intensive care unit.^10^ Furthermore, Adediji et al. demonstrated that aqueous extract of AN changes the blood levels of ALT, ALP, and AST and has detrimental effects on the liver of adult Wistar rats.^11^ In other studies, the AN was reported to be hepatotoxic, causing both cholestatic and hepatocellular hepatic injury and increased blood transaminases and alkaline phosphatase.^12^, ^13^ Considering the citation mentioned above, we propose that the observed elevation of liver enzymes in this case report is most likely due to both hepatic shock as a predisposing factor and the hepatotoxic effects of ANs.

Regarding the neurologic effects of the AN, it has been previously described that betel chewing causes widespread cortical desynchronization of the EEG, indicating a state of arousal, and betel nut‐induced extrapyramidal syndrome has also been well documented.^14^, ^15^ A betel nut‐induced seizure was reported in another case report in a young male. The patient had no other neuroradiologic or precipitating factors, and full recovery was observed without medication after abstinence.^16^ Although these reports were in line with our observation of our patient, a recent study found that the social practice of chewing Areca catechu nuts was linked to fewer seizure episodes in people with epilepsy. This observation could be due to the nut's natural stimulant properties or other reasons.^17^ This inconsistency should be investigated in future studies.

AN should be considered a cytotoxic and hepatotoxic agent. The patient's health conditions improved relatively rapidly after AN toxicity. In this case report, we concluded that the observed signs and symptoms, including decreased mental status, seizure, increased liver enzymes, and hemolysis, were due to AN's ingestion.

## AUTHOR CONTRIBUTIONS

**Seyede Maryam Mahdavi Mortazavi:** Data curation; visualization; writing – original draft. **Maryam Ataollahi:** Conceptualization; project administration; supervision; validation. **Amirali Mashhadiagha:** Conceptualization; writing – original draft. **Seyed Ali Moosavi:** Visualization; writing – original draft. **Reza Moshfeghinia:** Conceptualization; writing – original draft; writing – review and editing. **Marzieh Soheili:** Conceptualization; data curation; writing – original draft; writing – review and editing.

## FUNDING INFORMATION

The authors do not declare a specific grant for this research from any funding agency in the public, commercial or not‐for‐profit sectors.

## CONFLICT OF INTEREST STATEMENT

The authors declare that they have no competing interests.

## CONSENT STATEMENT

Written informed consent was obtained from the patient's legal guardian for publication of this case report. A copy of the written consent is available for review by the Editor‐in‐Chief of this journal.

## Data Availability

The data that support the findings of this study are from the corresponding author, Marzieh Soheili, upon reasonable request.
